# The Impact of Digital Health Interventions on Quality of Life Outcomes in Heart Failure Patients: A Systematic Review and Meta-Analysis

**DOI:** 10.7759/cureus.95964

**Published:** 2025-11-02

**Authors:** Uchenna Obonna

**Affiliations:** 1 Internal Medicine, St. George's University School of Medicine, London, GBR

**Keywords:** chronic heart failure, health related-quality of life, heart failure management programmes, remote monitoring, tele-health

## Abstract

Despite routine management, heart failure (HF) patients often contend with a burdensome symptomatology negatively affecting their quality of life (QoL). The use of digital health interventions (DHIs) such as mobile health (mHealth) applications, wearable devices, and remote monitoring systems (RMS) is transforming the management of cardiovascular disease (CVD). However, evidence regarding their effectiveness in improving QoL remains limited. This systematic review and meta-analysis aimed to evaluate the impact of DHIs on QoL in patients with HF.

A systematic search of randomised controlled trials (RCTs) and observational studies identified 14 studies evaluating QoL; nine of them reported statistically significant improvements associated with DHIs. Among the three studies assessing self-care outcomes, two demonstrated statistically significant improvements with DHI use, while one reported a non-significant reduction. Meta-analysis of six studies utilising the Minnesota Living with Heart Failure Questionnaire (MLHFQ) revealed a significant improvement in QoL (-8.06, 95% confidence interval (CI): -11.0 to -5.04), although this finding was limited by considerable heterogeneity (p=0.0025, I²=73%). Additionally, analysis of four studies employing the Kansas City Cardiomyopathy Questionnaire (KCCQ) indicated a statistically significant improvement in QoL (2.13, 95% CI: 1.34 to 2.92) with no significant heterogeneity (p=0.1719, I²=35.3%).

Overall, this review demonstrates that DHIs generally have a positive impact on QoL in HF patients. However, the heterogeneity within the meta-analysis due to the varied intervention types used, study durations, and participant characteristics highlights the need for more focused, high-quality research to facilitate stronger meta-analyses. Future studies should aim to assess the most effective components of DHI types, evaluate longer-term outcomes, and explore implementation in real-world settings.

## Introduction and background

Heart failure (HF), affecting over 64 million individuals worldwide [1], is a substantial burden on public health. It is characterised by a progressive impairment of cardiac function and physical capacity, culminating in repeated episodes of decompensation with frequent hospital admissions followed by limited recovery and a general overall decline in health, which ultimately leads to death [2]. Despite significant advances in both pharmacological and non-pharmacological therapies, HF patients continue to experience a substantial symptom burden that affects physical, psychological, and social domains [3]. Consequently, many individuals report a poor quality of life (QoL), a subjective measure encompassing an individual’s mental, physical, and emotional well-being, as well as social functioning and autonomy. Evidence indicates that patients with HF experience significantly poorer QoL compared to those with other chronic conditions [4], highlighting a need for improvement in HF management.

Routine HF management typically includes outpatient clinic visits and nurse-led programmes focused on patient education, lifestyle modification, and cardiac rehabilitation following discharge. Nevertheless, despite these well-established interventions, (QoL) outcomes in these patients remain suboptimal. Consequently, research has increasingly focused on the integration of technology-based health solutions that utilise digital tools to enhance traditional, clinic-based care, collectively referred to as digital health interventions (DHIs).

Emerging DHIs, such as mobile health (mHealth) applications, wearable devices, and remote monitoring systems (RMS), are transforming the management of cardiovascular disease (CVD), providing more personalised and continuous care. They enable continuous, real-time collection of physiological data, facilitate early detection of cardiac abnormalities, and promote active patient participation through self-management. The COVID-19 pandemic accelerated the adoption of DHI, highlighting its potential to address the limitations of conventional care and facilitate a more patient-centred approach to HF management [5]. Despite increasing interest and adoption of DHI, high-quality evidence supporting their clinical efficacy remains limited. Further research is needed to evaluate the effectiveness of various digital technologies and to guide their integration into routine HF care pathways, thereby improving patient QoL.

This systematic review and meta-analysis aimed to assess randomised controlled trials (RCTs) evaluating the clinical effectiveness of DHIs on QoL in HF patients. The primary objective was to consolidate current evidence regarding the impact of DHIs, thereby informing both future research and clinical practice.

## Review

Methods

Search Strategy

This systematic review was conducted by a single reviewer and included RCTs and observational/cohort studies published between January 1, 2015, and May 5, 2025, that examined any form of DHI (mobile phone applications, websites, text messaging, remote monitoring sensors, emails) and its impact on QoL within a study population of adults (≥18 years) with HF. Control interventions comprised usual care in accordance with standard guidelines or non-DHI interventions such as telephone calls. Exclusion criteria included protocol papers, studies assessing only usability or adherence, systematic reviews, studies that specifically enrolled patients with additional comorbidities, and those where the intervention targeted healthcare providers rather than patients.

**Figure 1:**
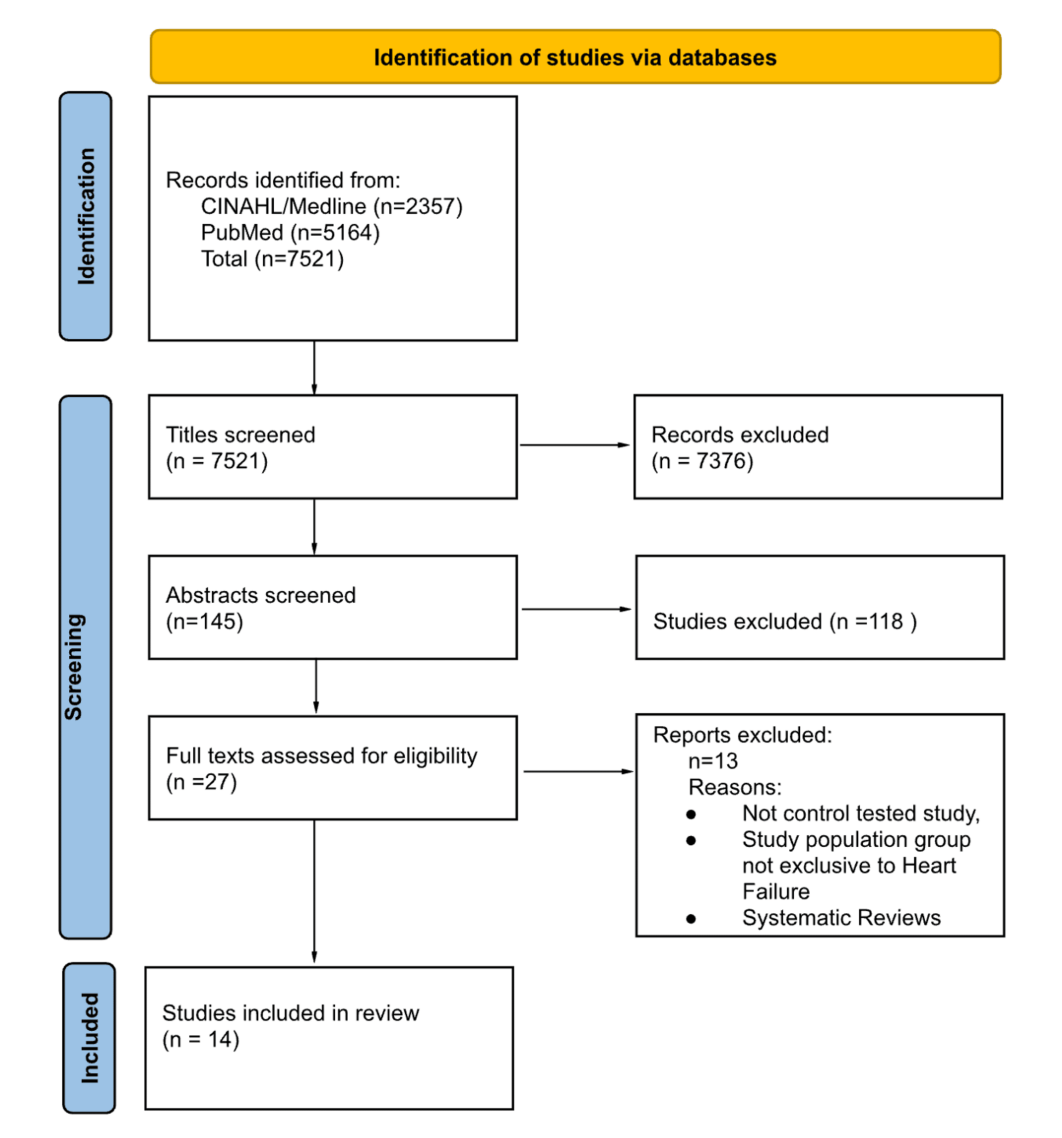
PRISMA flow diagram illustrating the study selection process Adhering to PRISMA 2020 guidelines, this flowchart illustrates how studies were selected for inclusion in this review. It shows the number of studies identified through database searches and other methods, the quantity of studies screened for eligibility, and the number of studies excluded at each stage. The chart also details the reasons for exclusions and indicates the final quantity included in the review. Each phase of the process is represented by labelled boxes connected by arrows to show the progression from initial identification to final inclusion PRISMA: Preferred Reporting Items for Systematic Reviews and Meta-Analyses

The search was conducted using the databases PubMed, MEDLINE, and CINAHL between the specified dates. The following keywords and Medical Subject Headings (MeSH) terms were included in the search: Digital Health, eHealth,mHealth, Telemedicine, Mobile Applications, Remote Consultation, Telehealth, Mobile Health Units, Wearable Electronic Devices, Wearable Technology, Biosensing Techniques, Sensors, Smartphone, Smartwatch, Remote Monitoring, Remote Sensing Technology, Remote Patient Monitoring, Telemetry, Home Monitoring, Cardiovascular Diseases, Heart Failure, Quality of Life. This search identified 145 studies for abstract screening. Figure 1 shows a detailed PRISMA (Preferred Reporting Items for Systematic Reviews and Meta-Analyses) flow diagram illustrating the search process.

Data Extraction and Risk of Bias Assessment

The data extracted includes the age and gender of control and intervention participants, the total number of participants, the type of DHI received, and its duration. The DHIs identified include mobile applications, websites, home-based rehabilitation with video-conferencing, remote monitoring sensors, and telemedicine. QoL outcomes include the European Heart Failure Self-care Behaviour Scale (EHFScB), MLHFQ (Minnesota Living with Heart Failure Questionnaire) score (Note that a lower score indicates better QoL), KCCQ (Kansas City Cardiomyopathy Questionnaire) Score, Self-Care Heart Failure Index (SCHFI) questionnaire score, SF-36 (Short Form Health Survey) Score, and EuroQol Score. This review excludes follow-up QoL score outcomes that were collected after completion of DHIs. Two identified studies provide QoL outcomes over multiple durations; in this case, outcomes provided over the shortest duration were extracted to minimise the effects of loss to follow-up. Risk of bias was assessed using the Cochrane Risk of Bias 2 (RoB 2) tool [6].

Data Synthesis and Analysis

Where possible, this review conducted meta-analyses to estimate treatment effects by pooling mean differences in QoL scores, using random-effects models with inverse variance weighting. Heterogeneity for each QoL outcome was assessed using the I² statistic. All analyses were performed using MetaAnalysisOnline.com [7].

To facilitate comparison and minimise heterogeneity, separate meta-analyses were performed for studies reporting KCCQ and MLHFQ scores, as there were sufficient numbers of studies available for each (four for KCCQ and six for MLHFQ). When standard deviations (SDs) were not reported, they were estimated from standard errors calculated using reported confidence intervals. In cases where confidence intervals (CIs) were unavailable, they were back-calculated using the reported mean differences and p-values.

As this review is based solely on data from previously published studies, ethical approval was not required.

Results

Risk of Bias, Characteristics, and Outcomes

A total of 14 studies were included in the final review following the screening process. A summary of the key characteristics of each study is presented in Table 1.

**Table 1:** Summary of studies included in final review This table provides a comprehensive summary of all the studies included in the final review. Each row corresponds to an individual study, with the columns outlining key characteristics such as the total number of participants, mean age (with standard deviation, where available), and the percentage of female participants and completion rate in both the intervention and control groups. Additional information includes the duration of the programme, the type of intervention implemented, and the main outcomes reported. Where available, the findings include mean differences in QoL scores with corresponding p-values, as well as mean differences in self-care scores and their respective p-values. For studies that included multiple intervention subgroups, the relevant outcomes and characteristics have been presented separately. Any data not reported by the study is indicated in the table using brackets. E-health: electronic health; EuroQol: heart failure symptom survey; ITT: intention to treat; QoL: quality of life; HCP: healthcare practitioner; KCCQ: Kansas City Cardiomyopathy Questionnaire; MD: mean difference; mHealth: mobile health; MLHFQ: Minnesota Living with Heart Failure Questionnaire; SF-36: The 36-Item Short Form Health Survey

Study	Total participants	Intervention participants (mean age in years, % female, % completed)						Control participants (mean age in years, % female, % completed)	Programme duration	Type of intervention	Result (QoL mean difference (MD), scoring system, p-value +/- CI						Result (self-care mean difference (MD) and p-value)	
Evagelista et al., 2015 [8]	42	21						21	3 months	Remote monitoring system with HCP virtual reviews and interventions	-7.9						N/A	
																			73.1 ± 9.2 years						72.3 ± 8.8 years
																										MLHFQ					
		47.6% female						47.6% female																							
											P<0.001																				
																			100%						100%						
		Nouryan et al., 2019 [9]	89	42																						47	6 months	Remote monitoring system with HCP virtual reviews and interventions	-6.1						N/A	
																																					81.4 years						84.9 years
											MLHFQ																																
				Female% not disclosed															Female% not disclosed																								
																				P=0.02																
																										100% (ITT used)						100% (ITT used)				
Cichosz et al., 2020 [10]	299			145						154	12 months	Virtual visits with self-monitoring of symptoms, vitals, and focus of self-empowerment + usual care	3.7						N/A																	
																																	70 years						69 years
																					KCCQ																		
				17% female						21% female																													
													P-value not significant (exact value not disclosed)																							
																													64.1%								64.9%
		Minh Tran et al., 2024 [11]	170	87						83											6 months	Telemedicine with remote monitoring at home with daily vitals, weight, and symptoms reported to HCPs with interventions	-14.2												N/A		
																																						60.9 ± 15.3 years						62.1 ± 14.7 years
													MLHFQ																															
				26.4% female						30.1% female																																		
																							P<0.0001													
																													N/A (group baseline analysis post follow-up)								N/A (group baseline analysis post follow-up)
Olivari et al., 2018 [12]	339			229						110	12 months	Remote monitoring system self-monitoring with vitals, symptoms, fluid status, and HCP virtual reviews of alarm values	Mental component			Physical component			N/A																		
				79.6 ± 6.8 years						80.9 ± 7.3 years			1.69			2.63																					
				38.9% female						34.6% female			SF-36 mental component			SF-36 physical component																				
				61.6%						57.3%			P<0.04			P<0.0001																				
Wagenaar et al., 2019 [13]	450			E-health intervention group			Website intervention group			150	3 months	Home monitoring on E-health website + usual care with feedback alerts (E-health)	E-health vs. control			Website vs. control			E-health vs. control	Website vs. control																
																							+7.4	+2.7												
		EHFScB	EHFScB																					
																					P<0.001	P<0.001		
																									66.6 ± 11 years			66.7 ± 10.4 years			66.9 ± 11.6 years	Intermediate intervention group: Home monitoring on the E-health website only (website group)	MLHFQ			MLHFQ		
																			32.7% female																				25.3% female		
																							27.3% female																		
		P<0.05																						P>0.05																	
																																										100% (ITT used)			100% (ITT used)		
																																																100% (ITT used)
																																																	95% CI (-9.76 to -0.53)			95% CI (-4.42 to 4.81)		
																																																							Mirshahi et al., 2024 [14]	50	22						23	6 weeks	Telehealth palliative care weekly webinars and WhatsApp group rehab activities	1.54						N/A	
																																																																										44.7 ± 11.9 years						50.3 ± 10.2 years
																																																																																	KCCQ					
																																																									48% female						32% female																							
																																																																		P<0.001																				
																											88%																																															92%												
																																																									Yanicelli et al., 2021 [15]	30	15																15	90 days	Mobile app facilitating home telemonitoring with measurement of vitals, patient education, alerts, and feedback + usual care	N/A						+20.59	
																																																																																						Mean age not disclosed						Mean age not disclosed
																											EHFScB																																																																	
					34% female							7% female																																																																																
													P=0.004																																																																						
		100%													100%																																																																				
								Dorsch et al., 2021 [16]	83	42						41	6 weeks	Mobile app with self-monitoring + usual care						-7.1						-6.5																																																					
																																																							60.2 ± 9 years						62 ± 9 years																						
		MLHFQ																																															SCHFI score																																		
										33% female						37% female																																																																			
																							P=0.04						P=0.4																																																						
																															88%																				87.8%																																
		Schmaderer et al., 2021 [17]	80	mHealth+ intervention group			mHealth only intervention group			27		3 months	Main intervention group (mHealth+): mHealth app self-monitoring, reminders for meds, education + nurse practitioner virtual visits. Intermediate intervention group (mHealth): Mhealth app and self-monitoring only	MD not available						N/A																															
																																							27									26			
																							EuroQol (heart failure symptom survey)																											
				Mean age not disclosed			Mean age not disclosed																						Mean age not disclosed																					
																														Female% not disclosed									Female% not disclosed		
														P-value not significant (exact value not disclosed, Cohen's d 0.32)																											
				96.2%			88.4%																																		
										Victoria-Castro et al., 2023 [18]													182	Bodyport		Conversa		Noom		44	90 days		Mobile apps: Conversa (self-management focused with feedback based on vitals and symptoms); Noom (app focused on self-management, live support from coaches using parameters; Bodyport (app focusing on personalised self-management monitoring vitals, symptoms, and fluid status, feedback, and patient education)	Noom		Bodyport		Conversa		N/A	
																																										46		46		46	
				59 years	3.1		1.8		1.8																																						
														59 years		62.5 years		62 years																													
																																																13% female	KCCQ		KCCQ		KCCQ	
																																																							19% female		16% feemale		21% female	
																																																													81.8%	P=0.06		P=0.31		P=0.73	
																																																																				84.7%		82.6%		82.6%	
		Piorowicz et al., 2015 [19]	131									75								56																																																						8 weeks	Home-based remote monitored cardiac rehabilitation with an exercise training plan	MD not available						N/A	
																																																																																				56.4 ± 10.9 years						60.5 ± 8.8 years
																																																																																											SF-36					
												15% female								5% female																																																																												
																								P-value not significant (exact value not disclosed)																																																																								
																																										100%																																		100%																				
												Hwang et al., 2017 [20]	53							24																																																									29	12 weeks	Home-based rehabilitation with videoconferencing	-7						N/A	
														68 ± 14 years																67 ± 11 years																																																									
																										MLHFQ																																																													
				21% female																11% female																																																																			
														P-value not significant (exact value not disclosed)																																																																									
																								95.8%										89.7%																																																					
		Felker et al., 2022 [21]	187	92																95										3 months					mHealth intervention involving a wearable device collecting step count and personalised text-message feedback with goals, medication adherence + usual care	5.5, KCCQ, p=0.009						N/A																																													
																																												60 ± 11 years																																											
														59 ± 11 years	KCCQ																																																																								
				33% female																																																																																			
																								37% female	P=0.009																																																														
																																		90.2%																																																					
				86.3%																																							

An overview of the assessed risk of bias for each study is provided in Table 2. Overall, the risk of bias was predominantly low. However, four studies raised some concerns due to missing outcome data, and one study was flagged for some concerns related to inadequate randomisation.

**Table 2:** Risk of bias assessment This table presents the risk of bias assessment for all studies included in the final review, conducted using the Cochrane Risk of Bias 2 (RoB 2) tool. Each column represents a specific domain within the RoB 2 framework, outlining the risk level assigned to each study for that domain

Study	Randomisation	Deviation from intended intervention	Missing outcome data	Measurement of outcome	Selection of the reported result	Overall risk
Evagelista et al., 2015 [8]	Some concerns	Low risk	Low risk	Low risk	Low risk	Low risk
Nouryan et al., 2019 [9]	Low risk	Low risk	Low risk	Low risk	Low risk	Low risk
Cichosz et al., 2020 [10]	Low risk	Low risk	Low risk	Low risk	Low risk	Low risk
Minh Tran et al., 2024 [11]	Low risk	Low risk	Low risk	Low risk	Low risk	Low risk
Olivari et al., 2018 [12]	Low risk	Low risk	Some concerns	Low risk	Low risk	Low risk
Wagenaar et al., 2019 [13]	Low risk	Low risk	Low risk	Low risk	Low risk	Low risk
Mirshahi et al., 2024 [14]	Low risk	Low risk	Some concerns	Low risk	Low risk	Low risk
Yanicelli et al., 2021 [15]	Low risk	Low risk	Low risk	Low risk	Low risk	Low risk
Dorsch et al., 2021 [16]	Low risk	Low risk	Some concerns	Low risk	Low risk	Low risk
Schmarderer et al., 2021 [17]	Low risk	Low risk	Low risk	Low risk	Low risk	Low risk
Victoria-Castro et al., 2023 [18]	Low risk	Low risk	Some concerns	Low risk	Low risk	Low risk
Piorowicz et al., 2015 [19]	Low risk	Low risk	Low risk	Low risk	Low risk	Low risk
Hwang et al., 2017 [20]	Low risk	Low risk	Low risk	Low risk	Low risk	Low risk
Felker et al., 2022 [21]	Low risk	Low risk	Low risk	Low risk	Low risk	Low risk

Intervention Features

The details of the interventions are outlined in Table 1. All digital health interventions (DHIs) were implemented in addition to usual care.

Five studies [8-12] utilised RMS, which involved patient self-measurement of physiological parameters such as weight, blood pressure, and heart rate. These data were remotely reviewed by healthcare professionals (HCPs), who then adjusted patient management accordingly. Similarly, the study by Wagenaar et al. [13] employed a web-based self-measurement platform that generated feedback alerts, prompting intervention by HF nurses. Mirshahi et al. [14] implemented a telehealth palliative care program that included weekly webinars and remote rehabilitation activities delivered via WhatsApp groups.

Four studies [15-18] employed mobile applications. Yanicelli et al. [15] and Dorsch et al. [16] used apps that supported daily self-monitoring and education, providing feedback alerts and tailored recommendations based on physiological data. Schmaderer et al. [17] utilised a similar app, enhanced with nurse practitioner virtual visits and medication reminders, to promote adherence. Victoria-Castro et al. [18] compared three mobile applications: Conversa and BodyPort, which focused on self-management supported by physiological and symptom-based feedback, and Noom, which provided live coaching support for behaviour change.

Two studies focused on digitally enhanced home-based cardiac rehabilitation. Piorowicz et al. [19] employed a supervised training program with real-time feedback via remote ECG monitoring, supplemented by psychological support as needed. Similarly, Hwang et al. [20] employed real-time exercise rehabilitation and education via videoconferencing. Felker et al. [21] used wearable technology to collect step-count data, delivering personalised feedback and goal setting via text messages, alongside medication reminders.

Clinical Outcomes

**Figure 2:**
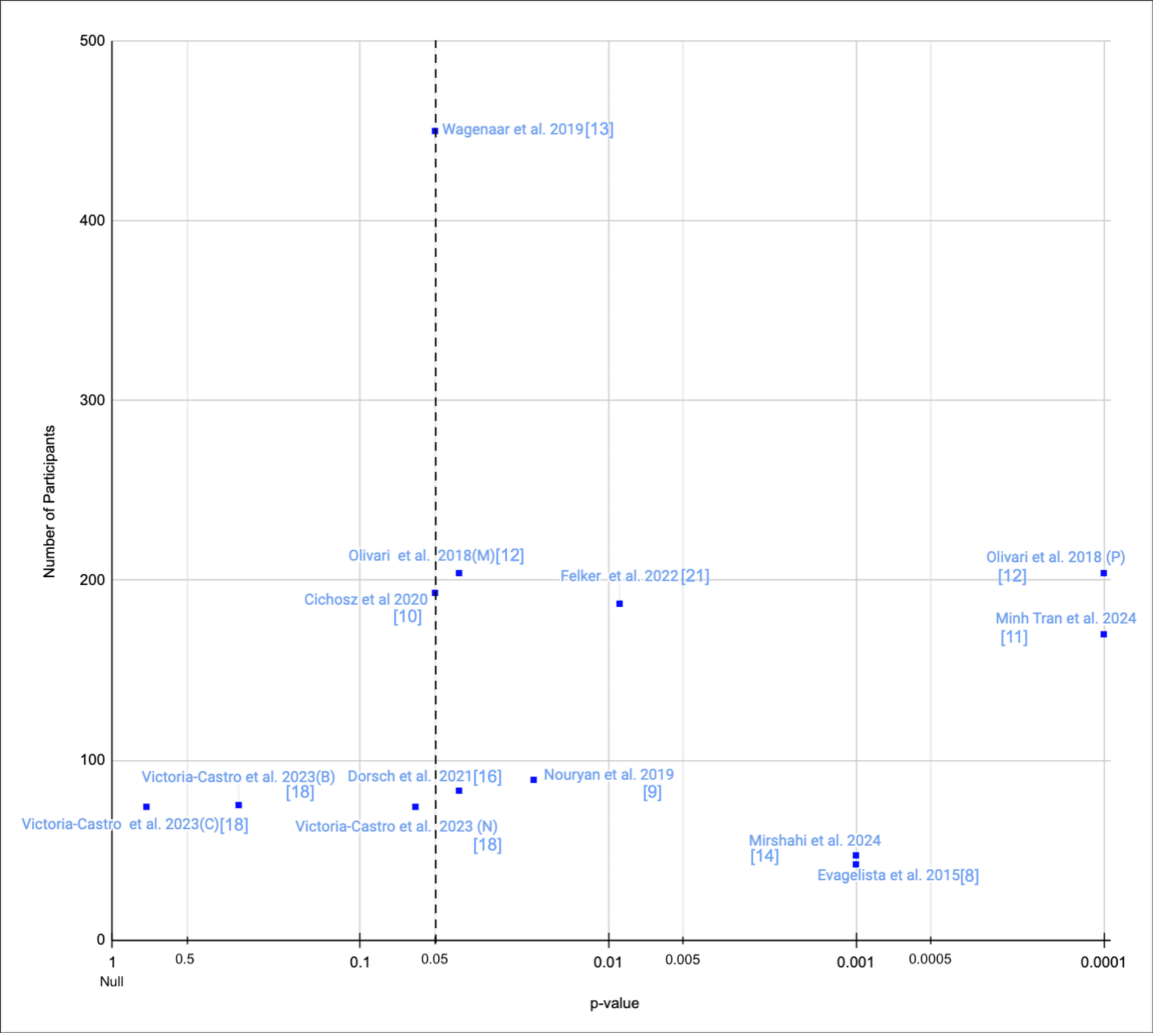
Scatter plot displaying p-values for mean diffenerce in QoL outcomes across included studies This plot presents each study’s reported p-value on the X-axis (with lower values indicating greater statistical significance, plotted toward the right), against the corresponding number of participants on the y-axis. A dotted vertical line marks the threshold for statistical significance (p=0.05). Studies that indicated a significant result (p<0.05) without specifying the exact p-value are plotted directly on this line. It is important to note that Victoria-Castro et al. [18] reported outcomes for three distinct DHIs, respectively, and each was illustrated separately in the scatter plot. Olivari et al. [12] reported p-values for mental component and physical component outcomes scores; these were both illustrated separately in the scatter plot. Note that all included studies reported a positive association, and so negative association p-values were not included on the X-axis QoL: quality of life; DHI: digital health intervention; C: Conversa DHI sub-group; N: Noom DHI sub-group; B: Bodyport DHI sub-group; M: mental component outcome; P: physical component outcome

Thirteen studies assessed QoL using various scoring systems: six used MLHFQ, four used the KCCQ, one used the EuroQol, and two used the SF-36. The mean differences and p-values, where reported, are summarised in Table 1. Figure 2 presents a scatter plot of the p-values from the included studies; four studies did not report p-values and are therefore excluded from the figure. All 13 studies included in Figure 2 demonstrated a positive association between DHI and QoL, with nine showing statistically significant improvements.

Given the demonstrated association in research between self-care and QoL scores [15], three studies [13,15,16] also assessed self-care outcomes. These were measured using the EHFScB and SCHFI scores. Yanicelli et al.'s [15] and Wagenaar et al.'s [13] studies showed statistically significant improvements in self-care with DHIs. Dorsch et al. [16] showed a mean reduction of -6.5 in the SCHFI score with no statistically significant change.

Meta-Analysis: The Effect of DHIs on QoL

**Figure 3:**
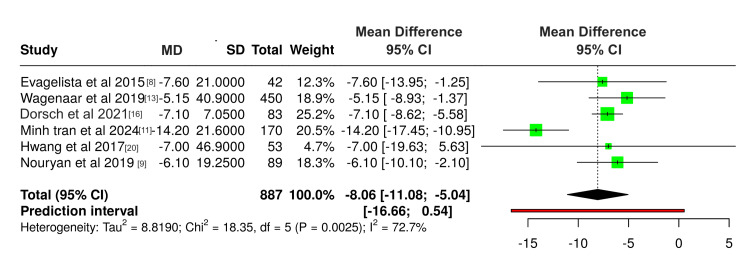
Forest plot showing the effect of DHI on QoL measured by MLHFQ DHI: digital health Intervention; QoL: quality of life; MLHFQ: Minnesota Living With Heart Failure Questionnaire; CI: confidence interval; MD: mean difference; SD: standard deviation

Group analysis of six studies measuring MLHFQ score (lower score indicates better QoL) showed a significant improvement of QoL (-8.06, 95% CI: -11.0; -5.04) (Figure 3); however, heterogeneity testing showed a p-value of 0.0025 and I^2 ^value of 73%, demonstrating a significant heterogeneity among the studies.

**Figure 4:**
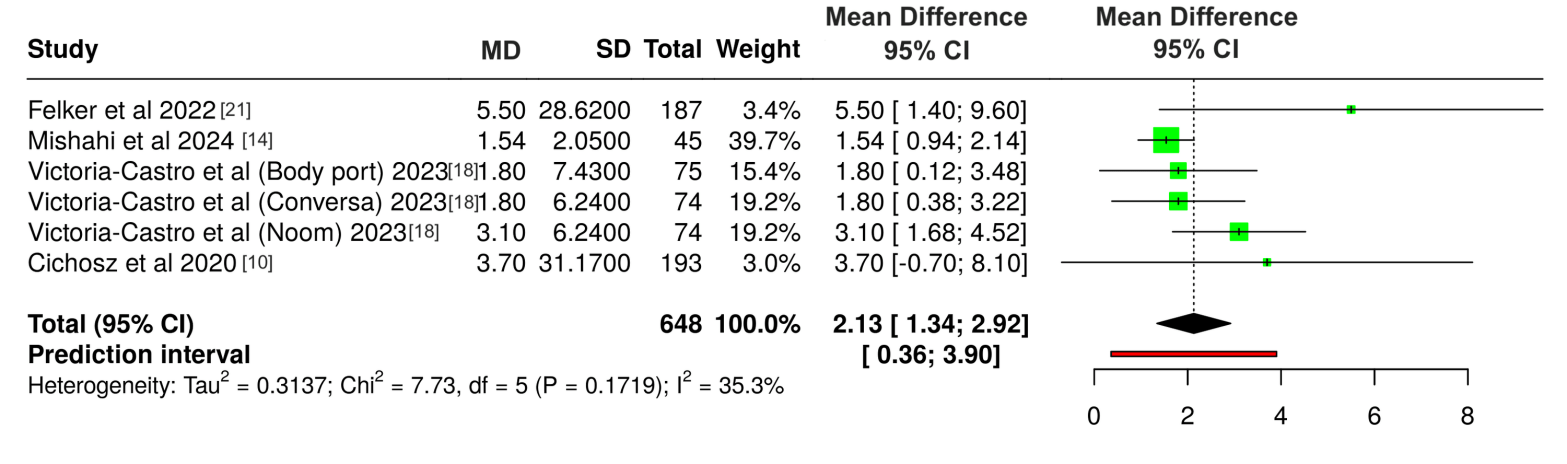
Forest plot showing the effect of DHI on QoL measured by KCCQ DHI: digital health Intervention; QoL: quality of life; KCCQ, Kansas City Cardiomyopathy Questionnaire; CI: confidence interval; MD: mean difference; SD: standard deviation

Group analysis of four studies measuring KCCQ score showed a statistically significant improvement in QoL (2.13, 95% CI: 1.34; 2.92) (Figure 4). Heterogeneity testing showed a p-value of 0.1719 and an I^2^ value of 35.3%, demonstrating no significant heterogeneity among the studies. It is important to note that Victoria-Castro et al. [18] reported outcomes for three distinct DHIs, and each was treated as a separate study in the analysis.

Discussion

The findings of this review demonstrate that DHIs generally have a positive impact on QoL outcomes in HF patients. All included studies reported improvements in QoL within their intervention groups, with nine out of 14 showing statistically significant improvements. QoL was assessed using various measurement tools, and a range of DHI types were implemented. The meta-analysis further supports this finding, showing a statistically significant reduction in MLHFQ scores among studies reporting this outcome. A similar positive effect was observed in studies measuring KCCQ scores.

Evidence of improvements in self-care was mixed. Two of the three studies [13,15] assessing this outcome reported statistically significant improvements. However, Dorsch et al. [16] found a non-significant mean reduction of 6.5 points in the SCHFI score in the intervention group, highlighting some variability in outcomes related to self-care. The authors attributed this result to higher baseline SCHFI scores among their participants compared to those typically reported in the HF literature. This may have been due to participants’ prior involvement in a similar telemanagement programme, potentially limiting the scope for further measurable improvement.

Although the existing literature on this topic is limited, previous studies have also demonstrated a positive impact of DHI on QoL outcomes. A recent systematic review examining the effect of telenursing on QoL outcomes analysed RCTs involving remote, nurse-led clinics that provided patient education and counselling via applications or telephone, compared to traditional face-to-face clinics [22]. The meta-analysis of eleven RCTs revealed a significant improvement in QoL outcomes; however, this was accompanied by substantial heterogeneity (I²=98%), likely due to variations in intervention durations and the QoL measurement tools used across different subgroups. Similarly, another systematic review assessing the impact of integrated palliative care telehealth in patients with chronic HF [23] reported a significant improvement in KCCQ scores. Notably, this review showed a lower level of heterogeneity (I²=46%) across the 16 included RCTs.

In this study’s meta-analysis, a mean reduction of 8.6 points in MLHFQ scores was observed with DHI, representing a clinically meaningful improvement. This is supported by previous literature [24], which considers a change of 5 points to be clinically significant. Given that the MLHFQ is scored out of a total of 105, with thresholds defining poor QoL as >45, good QoL as <24, and moderate QoL falling in between, the observed reduction further underscores the positive impact of DHI. Additionally, the DHI group showed a mean increase of 2.13 points in KCCQ scores. Although this reflects an improvement, the magnitude is relatively modest, as prior literature [25] indicates that a change of 5 points or more in the KCCQ is typically regarded as clinically meaningful.

Limitations

The broad inclusion criteria of this review allowed for the incorporation of studies employing diverse DHI modalities across varied HF patient populations, encompassing differences in age, socioeconomic status, clinical condition, and intervention duration. This diversity contributed to the substantial heterogeneity observed in the MLHFQ meta-analysis. Despite this, the analysis still demonstrated an overall positive effect of DHIs on QoL. However, the heterogeneity across the study sample populations and in DHI types limits the strength and generalisability of the conclusions drawn.

Several studies had small sample sizes, with four enrolling fewer than 53 participants, potentially affecting the strength of their findings. Moreover, this review did not assess factors related to the usability or accessibility of DHIs, which will likely influence intervention uptake and effectiveness in real-world settings. There is also a risk of publication bias, as only published studies were included. Additionally, four studies were rated as having “some concerns” for risk of bias due to missing outcome data, and one study due to issues with randomisation procedures. It should also be noted that over-representation of English language publications within the CINAHL, PubMed, and MEDLINE databases introduces a language bias to the publication search of this review. Finally, due to time and resource constraints, the screening process was conducted by a single reviewer. There is, therefore, a potential risk of selection bias, which may have impacted the inclusion and exclusion of studies.

Future research directions

Currently, there is limited high-quality research on the impact of DHI on QoL in HF patients. To strengthen the evidence for DHI use, future studies should prioritise several areas. The consistent use of standardised QoL measures in future research, such as MLHFQ and KCCQ, is essential to improve comparability and support meta-analyses. As most RCTs are short-term, a longer follow-up is needed to assess the durability of DHI effects. Further research should involve more focused assessments on which components of DHI (e.g., remote monitoring, education, self-management support) contribute most to QoL improvements and whether intervention intensity influences outcomes. Studies analysing the impact of factors such as age, HF severity, digital literacy, and socioeconomic status on outcomes are important to guide the application of personalised care. In addition, cost-effectiveness studies are needed to evaluate DHI’s cost relative to standard care. Finally, research into the implementation of DHI is needed to explore real-world barriers and facilitators for its use. These could focus on feasibility, adherence, and scalability.

## Conclusions

This review demonstrates that DHIs generally exert a positive effect on the QoL of HF patients. Most studies reported improvements in QoL, with a significant proportion showing statistically significant changes. Additionally, clinically meaningful changes were found in MLHFQ score analysis. Although the effect on self-care outcomes was mixed, the overall findings support the use of DHI in HF management. However, the heterogeneity within the meta-analysis due to varied DHI types used, participant characteristics, and study durations demonstrates the need for more focused, high-quality research to facilitate stronger meta-analyses. Future studies should aim to assess the most effective components of DHI types, evaluate longer-term outcomes, and explore implementation in real-world settings.
